# Use of Aztreonam–Avibactam with Rapid Eravacycline Step-Down Therapy for a Tibial Septic Non-Union by NDM-Producing *Enterobacter cloacae*

**DOI:** 10.3390/antibiotics14111109

**Published:** 2025-11-04

**Authors:** Jacob M. Keck, Ryan K. Dare, Michael Saccente, Keyur S. Vyas, Rebekah N. Thompson

**Affiliations:** 1 Department of Pharmacy, University of Arkansas for Medical Sciences (UAMS), Little Rock, AR 72205, USA; 2 Department of Medicine, Division of Infectious Diseases, University of Arkansas for Medical Sciences (UAMS), Little Rock, AR 72205, USA

**Keywords:** hardware infection, New Delhi metallo-β-lactamase (NDM)-producing *Enterobacterales*, aztreonam-avibactam, eravacycline, cefiderocol

## Abstract

New Delhi metallo-β-lactamase (NDM)-producing *Enterobacterales* represent a major therapeutic challenge due to their resistance to nearly all β-lactams and frequent co-resistance to other antibiotic classes, leaving clinicians with few effective options. These challenges are amplified in orthopedic infections with hardware involvement, where biofilm formation and the need for prolonged antimicrobial therapy limit success. We describe a 55-year-old female with a history of right type 3 open pilon fracture complicated by hardware failure and revision, who presented with septic tibial nonunion and chronic drainage. During this admission, she underwent irrigation and debridement with hardware removal and intramedullary nail placement. Cultures grew *Enterobacter cloacae* complex resistant to meropenem, ceftazidime–avibactam, meropenem–vaborbactam, and cefiderocol, as well as *Candida parapsilosis*. Molecular testing confirmed NDM production, while reference testing showed susceptibility to aztreonam–avibactam (ATM-AVI). The patient was treated with ATM-AVI plus micafungin, achieving clinical stability within three days. Due to outpatient administration barriers with ATM-AVI, the patient was transitioned to eravacycline and micafungin. At eight-week follow-up, the patient remained clinically improved without relapse or adverse effects. This case highlights ATM-AVI as a critical therapy for NDM-producing orthopedic infections involving hardware and supports eravacycline as a feasible step-down option in outpatient management.

## 1. Introduction

The global rise of antimicrobial resistance (AMR) continues to reduce the effectiveness of existing therapies, creating profound challenges in the management of multidrug-resistant (MDR) infections [1]. Among the most formidable threats are infections caused by New Delhi metallo-β-lactamase (NDM)-producing *Enterobacterales*, which exhibit intrinsic resistance to nearly all β-lactam agents and frequently co-harbor additional resistance determinants against aminoglycosides, fluoroquinolones, and even last-line therapies such as colistin [2,3,4]. This convergence of resistance mechanisms leaves clinicians with few viable options, often forcing reliance on agents with uncertain efficacy or substantial toxicity [5]. The consequences are substantial, including higher rates of clinical failure, prolonged hospitalizations, and increased morbidity and mortality [5,6,7,8]. These realities underscore the pressing need for novel antimicrobial agents capable of reliably overcoming metallo-β-lactamase (MBL)-mediated resistance and improving patient outcomes.

The management of orthopedic infections due to MDR organisms presents additional complexities beyond those encountered in systemic infections [9,10,11]. Optimal care requires a multidisciplinary approach that integrates prolonged antimicrobial therapy with surgical strategies, including incision and debridement, hardware retention or exchange, and staged revision procedures [12,13,14,15]. When MDR pathogens such as NDM-producing *Enterobacterales* are involved, therapeutic options become exceedingly limited, complicating both curative and suppressive approaches [16,17]. Biofilm formation on prosthetic material further reduces antibiotic penetration and efficacy, heightening the risk of persistent infection and relapse [10,18]. These clinical realities emphasize the urgent need for agents with reliable activity against MDR pathogens and predictable pharmacokinetic and pharmacodynamic characteristics to ensure adequate drug delivery in the setting of orthopedic infection.

Cefiderocol, a siderophore cephalosporin, initially generated enthusiasm given its stability against MBL enzymes and broad in vitro spectrum [19,20]. However, increasing reports of both acquired and intrinsic resistance among NDM-producing organisms have tempered confidence in its utility [21,22,23,24,25,26,27,28]. Resistance has been observed to emerge during treatment and has been documented even in the absence of prior exposure, particularly in isolates carrying NDM-1 and NDM-5 [21,23,24,29]. These findings are concerning given that cefiderocol was considered one of the few viable therapeutic options against MBL-producing Enterobacterales. Consequently, dependence on cefiderocol as a cornerstone therapy is increasingly precarious, further narrowing an already limited armamentarium. In contrast, aztreonam-avibactam (ATM-AVI) has emerged as one of the most promising next-generation β-lactam/β-lactamase inhibitor combinations, especially for treating infections involving NDM-producing pathogens [30,31,32,33]. In the pivotal REVISIT phase 3 trial, ATM-AVI demonstrated noninferiority to meropenem for serious Gram-negative infections, including hospital-acquired and ventilator-associated pneumonia, complicated intra-abdominal infections, and bloodstream infections. Of particular importance, patients with MBL-producing *Enterobacterales*, including NDM, achieved favorable clinical and microbiological outcomes, highlighting ATM-AVI’s unique therapeutic potential in this high-risk population [34].

Here, we present a case of septic nonunion due to an NDM-producing *Enterobacter cloacae*, successfully managed with ATM-AVI therapy and with rapid transition to eravacycline. This case illustrates both the challenges and evolving therapeutic strategies for MDR orthopedic infections, while underscoring the importance of expanding effective treatment options for infections driven by MBL-mediated resistance. Furthermore, this case report adds to the limited literature on orthopedic nonunion infections involving MBL enzymes, specifically NSDM, as well as the underreported utilization of ATM-AVI and eravacycline for orthopedic infections involving MDR pathogens.

## 2. Patient Case

A 55-year-old female with a history of a right type III open pilon fracture occurring in December 2023 underwent external fixation with a spatial frame. The patient subsequently developed hardware loosening and required revision surgery several weeks later. No additional surgical procedures were performed; however, the patient reported intermittent drainage from the surgical site for over a year following the hardware revision. In January 2025, the patient was diagnosed with tibial septic non-union and underwent surgical debridement with hardware removal and intramedullary nail placement. Intraoperative cultures grew methicillin-resistant *Staphylococcus aureus* (MRSA) and completed a six-week course of daptomycin combined with rifampin.

In August 2025, the patient was readmitted with a chronic draining wound, exposed bone, and implant. At that time, surgical debridement was performed with removal of deep implants, debridement of infected bone, and revision fixation to address the septic nonunion. Two days postoperatively, intraoperative cultures yielded *Enterobacter cloacae* complex resistant to meropenem (MIC > 16 µg/mL) and *Candida parapsilosis* susceptible to both micafungin and fluconazole. Additional susceptibility testing for the *Enterobacter cloacae* complex demonstrated resistance to ceftazidime-avibactam, meropenem-vaborbactam, and cefiderocol, despite no prior cefiderocol exposure (Table 1). The patient was initiated on aztreonam–avibactam (ATM-AVI) 2000 mg every 6 h plus micafungin 150 mg daily. Molecular testing using Cepheid Xpert^®^ CARBA-R confirmed the presence of an NDM carbapenemase, and reference laboratory testing confirmed susceptibility to ATM-AVI (Table 1).

Three days after initiation of ATM-AVI, the patient remained clinically stable and was prepared for discharge. Due to the cost and logistical challenges of prolonged outpatient ATM-AVI infusions, the patient was transitioned to eravacycline 1.5 mg/kg daily plus micafungin 150 mg daily, which was later switched to fluconazole 400 mg daily because of insurance limitations. The treatment plan included six weeks of eravacycline, followed by oral omadacycline for suppressive therapy, along with a three-month course of micafungin/fluconazole. At the eight-week follow-up, the patient demonstrated continued clinical improvement, with resolution of orthopedic symptoms and no adverse drug effects. Suppressive therapy with oral omadacycline 300 mg daily was maintained due to retained hardware, and there was no evidence of infection recurrence at three months post-debridement. 

## 3. Discussion

This case underscores the escalating challenge of managing orthopedic infections in the era of rising AMR. Infections involving orthopedic hardware are notoriously difficult to eradicate due to biofilm formation, impaired vascular supply, and the frequent need for staged surgical interventions. The presence of MDR Gram-negative organisms further complicates therapy, often requiring prolonged intravenous regimens with agents that may offer limited efficacy, significant toxicity, or logistical barriers. Our patient, a 55-year-old female with a complex orthopedic history, experienced multiple hardware revisions and chronic wound drainage beginning in late 2023. The patient presented in January 2025 with a septic right tibial nonunion following several prior interventions and was treated a six-week course of daptomycin combined with rifampin for methicillin-resistant *Staphylococcus aureus* (MRSA). In August 2025, the patient required another debridement and removal of deep implants secondary to chronic draining wounds. Intraoperative cultures at this time grew *Enterobacter cloacae* complex resistant to meropenem, ceftazidime–avibactam, meropenem–vaborbactam, and cefiderocol, along with *Candida parapsilosis*. Molecular testing confirmed NDM gene expression, and notably, cefiderocol resistance emerged despite no prior exposure. The patient was initiated on ATM-AVI plus micafungin inpatient, resulting in clinical stabilization. Due to the practical challenges of ATM-AVI outpatient administration (every 6 h over 3 h with no continuous infusion data available), the patient was transitioned to eravacycline based on susceptibility results plus micafungin (later switched to fluconazole). After six-weeks of therapy, the patient showed sustained clinical improvement without any adverse drug effects, highlighting both the challenges and the potential for successful management of highly resistant orthopedic infections. The patient was subsequently transitioned to suppressive therapy with omadacycline and remained clinically stable at the three-month follow-up.

Reports of NDM-producing orthopedic infections, including prosthetic joint infections (PJI), remain scarce. Rubnitz and colleagues described a 39-year-old patient with recurrent osteomyelitis after intramedullary tibial nail placement; cultures revealed NDM-producing *Citrobacter sedlakii*. Initial eravacycline therapy failed, but a subsequent six-week course of ceftazidime-avibactam plus aztreonam achieved microbiologic eradication and clinical resolution despite retained hardware [35]. Similarly, Santana et al. reported chronic PJI complicated by NDM-producing *Klebsiella pneumoniae* and ESBL-producing *Enterobacter cloacae*, managed successfully with hardware removal, abscess debridement, and combination therapy including ceftazidime-avibactam, aztreonam, rifampin, and polymyxin-B [17]. These reports underscore the clinical utility of combination of avibactam and aztreonam based regimens, with or without adjunctive agents, in achieving successful outcomes for NDM or other carbapenem-resistant *Enterobacterales* (CRE) infections, while highlighting the urgent need for further studies to define optimal treatment strategies.

Historically, cefiderocol was considered a dependable option against NDM-producing organisms; however, clinical experience shows variable efficacy and the potential for resistance [19,22,23,24,27,28]. In our patient case, cefiderocol resistance was noted without previous exposure. We hypothesis this likely reflects a combination of resistance mechanism including the production of NDM-1 or NDM-5, mutations in the genes encoding siderophore receptors leading to truncated iron-binding proteins, efflux pumps, and/or changes in penicillin binding proteins (PBP), specifically PBP-3, but confirmation via whole genome sequencing was not completed for the *Enterobacter cloacae* isolate [5,21,22,25,36]. In contrast, ATM-AVI represents a more reliable therapeutic option, combining aztreonam, intrinsically stable against MBL, including NDM, with avibactam, which inhibits co-produced serine β-lactamases commonly encountered in clinical practice [5,30,37,38,39]. This combination restores activity against organisms producing both MBL and serine β-lactamases and offers practical advantages as a single-agent regimen compared with dual-therapy approaches such as aztreonam plus ceftazidime–avibactam [38,39]. Supporting this, the phase 3 REVIST trial demonstrated that ATM-AVI was noninferior to meropenem for serious Gram-negative infections, including favorable outcomes in patients with MBL-producing *Enterobacterales* [34]. Surveillance studies also indicate >99% in vitro inhibition of *Enterobacterales*, including strains co-harboring NDM with other carbapenemases, while cefiderocol shows lower susceptibility in NDM-positive isolates [38,39]. Despite this, practical limitations for outpatient administration remain a barrier, as ATM-AVI typically requires four prolonged IV infusions daily, complicating its use in this setting [30,40].

Given ATM-AVI barriers with outpatient administration, alternative step-down therapies must be explored for MDR, including NDM, orthopedic infections. Eravacycline, a fully synthetic fluorocycline, exhibits enhanced activity against MDR Gram-negative and Gram-positive pathogens, including many NDM-producing strains [41,42,43,44,45,46,47,48]. Compared with tigecycline, eravacycline achieves higher serum concentrations, improved tissue penetration, and lower rates of gastrointestinal toxicity [49,50]. Although clinical data for eravacycline outside of skin and soft tissue, pulmonary, and intra-abdominal infections are limited, its use in hardware-associated infections is supported by favorable bone penetration and a low incidence of long-term adverse effects [51,52,53]. The successful use of eravacycline in this patient reinforces its potential role as a salvage or consolidation therapy in complex orthopedic infections.

While this case highlights the therapeutic use of ATM-AVI and eravacycline in managing a complex orthopedic infection, several limitations should be acknowledged. First, the relatively short follow-up period limits assessment of long-term clinical outcomes, particularly in infections involving retained hardware. Second, the rapid transition from ATM-AVI to eravacycline makes it difficult to attribute sustained clinical success solely to the initial regimen. Nonetheless, the decision to empirically initiate ATM-AVI based on genotypic confirmation of NDM underscores both the emerging concern of cefiderocol resistance and the clinical value of rapid molecular diagnostics in guiding early therapy [22,54]. Moving forward, integrating local and national AMR surveillance data with genotypic testing will be essential for optimizing treatment strategies in orthopedic infections caused by MDR pathogens.

In summary, this case underscores the critical importance of incorporating novel agents such as ATM-AVI into multidisciplinary treatment strategies for MDR orthopedic infections, while also exploring the complementary role of agents like eravacycline for extended therapy. It further highlights the limitations of current therapeutic options, particularly the diminishing efficacy of cefiderocol against NDM-producing pathogens and reinforces the need for ongoing clinical studies to refine treatment algorithms. The favorable clinical outcome observed in this case illustrates both the promise of emerging antimicrobial therapies and the persistent challenges clinicians encounter in managing complex orthopedic infections amid escalating resistance.

## 4. Conclusions

Orthopedic infections, especially those with retained hardware, caused by NDM-producing *Enterobacterales* present an escalating therapeutic challenge marked by extensive antimicrobial resistance and severely limited treatment options. This case illustrates the potential utility of ATM-AVI as an effective initial therapy for MBL-producing pathogens, even in the setting of cefiderocol resistance, and highlights the feasibility of eravacycline as a practical step-down option for prolonged management. The integration of novel β-lactam/β-lactamase inhibitor combinations and next-generation tetracyclines into multidisciplinary treatment strategies may enhance outcomes in these high-risk infections, particularly when conventional agents fail to achieve clinical success. Continued surveillance and prospective clinical studies are essential to define optimal therapeutic approaches, evaluate long-term efficacy, and support antimicrobial stewardship in the use of these emerging agents.

## Figures and Tables

**Table 1:** *Enterobacter cloacae complex* isolate susceptibilities and Cepheid Xpert^®^ CARBA-R.

Drug	MIC (mcg/mL)	Interpretation (CLSI/FDA Breakpoints)
Ceftazidime	>32	Resistant (CLSI)
Cefepime	>32	Resistant (CLSI)
Piperacillin-tazobactam	>128	Resistant (CLSI)
Meropenem	>16	Resistant (CLSI)
Levofloxacin	>8	Resistant (CLSI)
Gentamicin	8	Resistant (CLSI)
Ceftazidime-avibactam	>16	Resistant (CLSI)
Meropenem-vaborbactam	>16/8	Resistant (CLSI)
Tigecycline	<0.5	Susceptible (FDA)
Eravacycline	0.25	Susceptible (FDA)
Omadacycline	4	Susceptible (FDA)
Cefiderocol	16	Resistant (CLSI)
Aztreonam-avibactam	0.125	Susceptible (FDA)
**Cepheid Xpert^®^ CARBA-R**		
Genotype	Result	Interpretation
CTX-M	NEG	Not expressed
KPC	NEG	Not expressed
OXA-48	NEG	Not expressed
NDM	POS	Expressed

Abbreviations: CTX-M (Cefotaximase-Munich); mcg (micrograms); MIC (minimal inhibitory concentration); mL (milliliter) NDM (New Delhi metallo-β-lactamase); NEG (negative); KPC (*Klebsiella pneumoniae* carbapenemase); OXA-48 (oxacillinase-48); POS (positive).

## Data Availability

No new data were created or analyzed in this study. Data sharing is not applicable to this article.
